# Supplementary material for: Warm Arctic episodes linked with increased frequency of extreme winter weather in the United States
Source: Nat Commun. 2018 Mar 13;9:869. doi: 10.1038/s41467-018-02992-9 (PMC5849726; doi:10.1038/s41467-018-02992-9)
Supplement: Supplementary file 1 — Supplementary Information [file 41467_2018_2992_MOESM1_ESM.pdf]

Supplementary Table 1. Statistical significance for difference in AWSSI between positive and negative PCH. Values that exceed 95% confidence level are shown in bold. Values were detrended and significance testing accounted for autocorrelation.

P-values: Polar Cap Height – Accumulated Winter Seasonal Severity Index												
	Level (hPa)											
	100	150	200	250	300	400	500	600	700	850	925	1000
ATLANTA HARTSFIELD INTL AP (GA)	**0**	**0**	**0**	**0**	**0**	**0**	**0**	**0**	**0**	**0**	**0**	**0**
BISMARCK (ND)	**0**	**0**	**0**	**0**	**0**	**0**	**0**	**0**	**0**	**0**	**0**	**0**
CHICAGO AREA (IL)	**0**	**0**	**0**	**0**	**0**	**0**	**0**	**0**	**0**	**0**	**0**	**0**
E MILTON BLUE HILL OBSY (MA)	**0**	**0**	**0**	**0**	**0**	**0**	**0**	**0**	**0**	**0**	**0**	**0**
DES MOINES AREA (IA)	**0**	**0**	**0**	**0**	**0**	**0**	**0**	**0**	**0**	**0**	**0**	**0**
DETROIT AREA (MI)	**0**	**0**	**0**	**0**	**0**	**0**	**0**	**0**	**0**	**0**	**0**	**0**
DULUTH INTL AP (MN)	**0**	**0**	**0**	**0**	**0**	**0**	**0**	**0**	**0**	**0**	**0.000006**	**0.000033**
HELENA AREA (MT)	**0**	**0**	**0**	**0**	**0**	**0**	**0**	**0**	**0**	**0**	**0**	**0.000001**
PHILADELPHIA INTL AP (PA)	**0**	**0**	**0**	**0**	**0**	**0**	**0**	**0**	**0**	**0**	**0**	**0**
SALT LAKE CITY (UT)	0.263697	0.232088	0.622911	0.082232	**0.01469**	**0.03196**	**0.03522**	**0.043813**	**0.021421**	**0.008901**	**0.005626**	**0.008003**
SEATTLE AREA (WA)	**0.020585**	**0.000438**	**0.000001**	**0.000258**	**0.000095**	**0.000039**	**0.000009**	**0.00004**	**0.001906**	**0.001682**	**0.006049**	**0.031757**
WASHINGTON REAGAN NATL AP (VA)	**0**	**0**	**0**	**0**	**0**	**0**	**0**	**0**	**0**	**0.000006**	**0.000037**	**0.000176**

Supplementary Table 2. Statistical significance for difference in AWSSI between positive and negative PCT. Values that exceed 95% confidence level are shown in bold. Values were detrended and significance testing accounted for autocorrelation.

P-values: Polar Cap Temperature – Accumulated Winter Seasonal Severity Index												
	Level (hPa)											
	100	150	200	250	300	400	500	600	700	850	925	1000
ATLANTA HARTSFIELD INTL AP (GA)	**0.000122**	**0.000008**	**0.000035**	**0.000016**	**0.000001**	**0**	**0.000003**	**0.000116**	**0.000266**	**0.002257**	**0.004687**	**0.004686**
BISMARCK MUNI (ND)	0.06487	**0.00154**	**0.003197**	**0.0003**	**0**	**0**	**0**	**0**	**0**	**0**	**0**	**0**
CHICAGO AREA (IL)	**0.015941**	**0.000707**	**0.001462**	**0.000351**	**0**	**0**	**0**	**0**	**0**	**0**	**0**	**0**
E MILTON BLUE HILL OBSY (MA)	**0.024012**	**0.000533**	**0.006373**	**0.000032**	**0**	**0**	**0**	**0**	**0**	**0**	**0**	**0**
DES MOINES AREA (IA)	0.102193	**0.003153**	**0.031234**	**0.019438**	**0.000005**	**0**	**0**	**0**	**0**	**0**	**0**	**0**
DETROIT AREA (MI)	**0.00524**	0.091027	**0.036422**	0.348535	**0.000713**	**0**	**0**	**0**	**0**	**0**	**0**	**0**
DULUTH INTL AP (MN)	0.850287	0.066622	0.085025	**0.027142**	**0.000004**	**0**	**0**	**0**	**0**	**0**	**0.000001**	**0.000047**
HELENA AREA (MT)	0.351161	0.462496	0.071482	0.760828	**0.000738**	**0**	**0**	**0**	**0**	**0**	**0**	**0**
PHILADELPHIA INTL AP (PA)	**0.021958**	**0.001792**	**0.038281**	**0.002494**	**0**	**0**	**0**	**0**	**0**	**0**	**0**	**0**
SALT LAKE CITY (UT)	0.513326	0.088079	0.214734	0.999356	0.491702	**0.010507**	**0.003691**	**0.046014**	0.080285	0.896799	0.119038	**0.023708**
SEATTLE AREA (WA)	0.731185	0.910728	0.997867	0.15237	0.020228	0.127821	**0.005339**	**0.000208**	**0.000178**	**0.000001**	**0**	**0**
WASHINGTON REAGAN NATL AP (VA)	**0.007067**	**0.000636**	**0.006339**	**0.002088**	**0**	**0**	**0**	**0**	**0**	**0**	**0**	**0.000001**

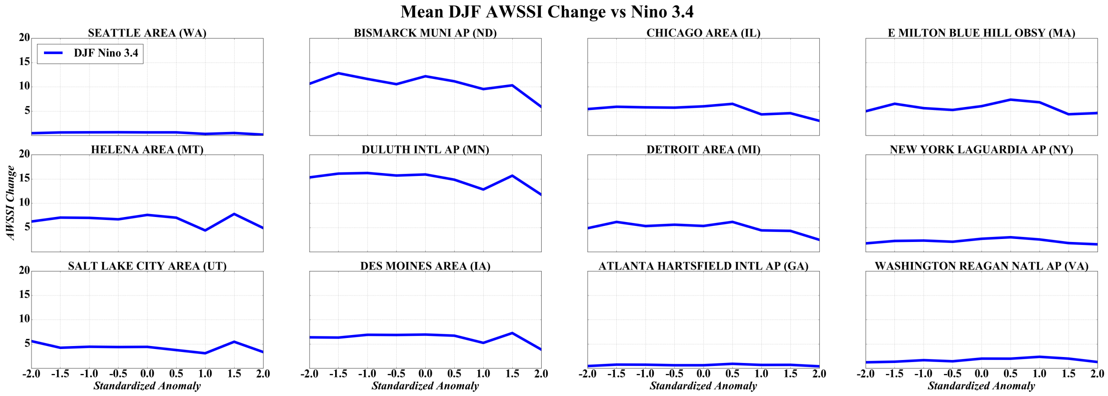

Supplementary Figure 1. The severe winter weather index is mostly insensitive to ENSO. The average daily change in the AWSSI at selected weather stations across the US associated with the 3.4 Niño index (the most common index used to classify El Niño and La Niña events) 1950-2016.

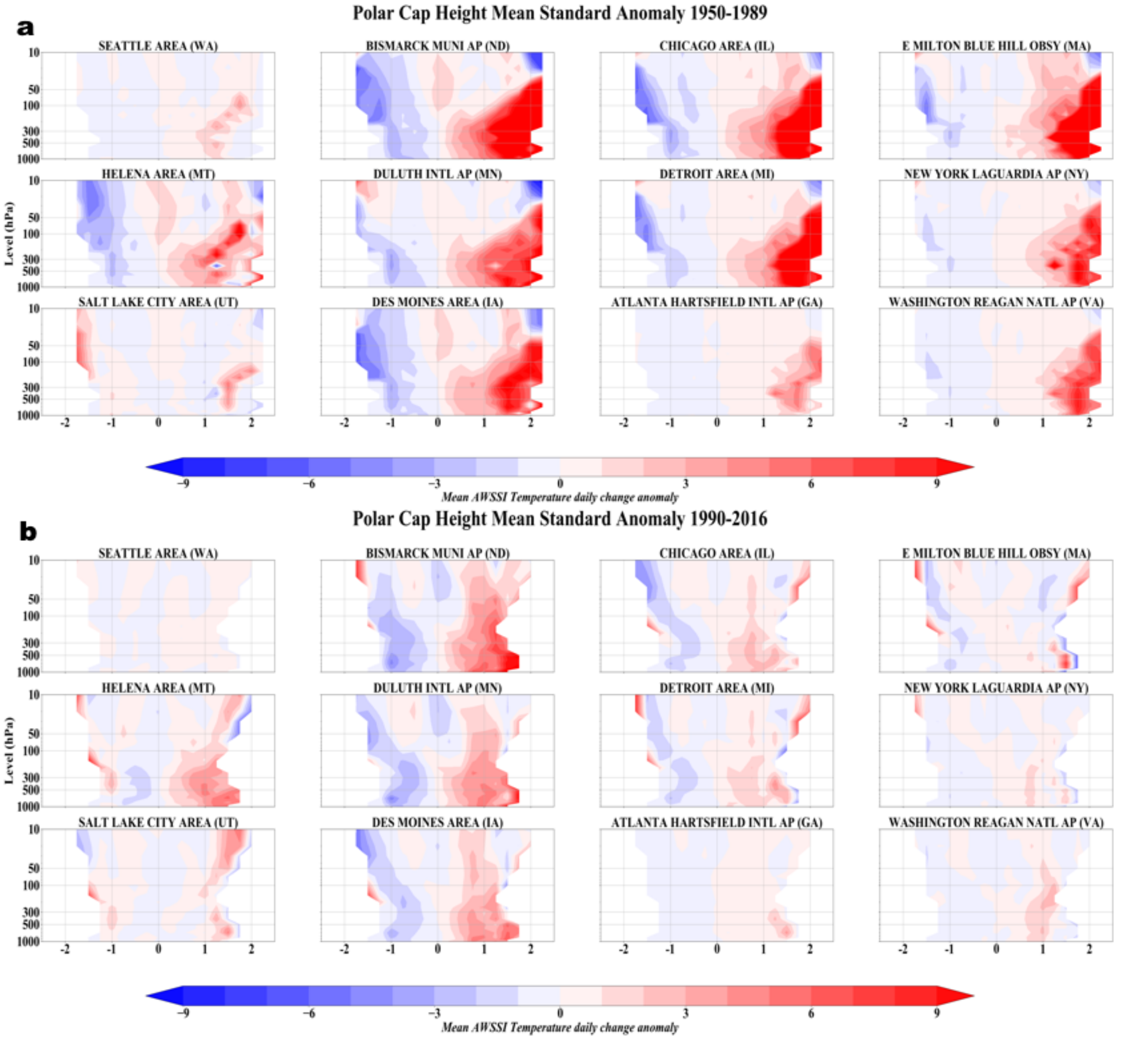

Supplementary Figure 2. Correlation between Arctic height anomalies and severe winter weather before and during period of Arctic Amplification. The average daily change in the AWSSI at weather stations across the US composited for all PCH values from the surface to the mid-stratosphere during years **a** with a relatively cold Arctic 1950-1989 and **b** a relatively warm Arctic 1990-2016. Anomalies computed relative to 1981-2010.

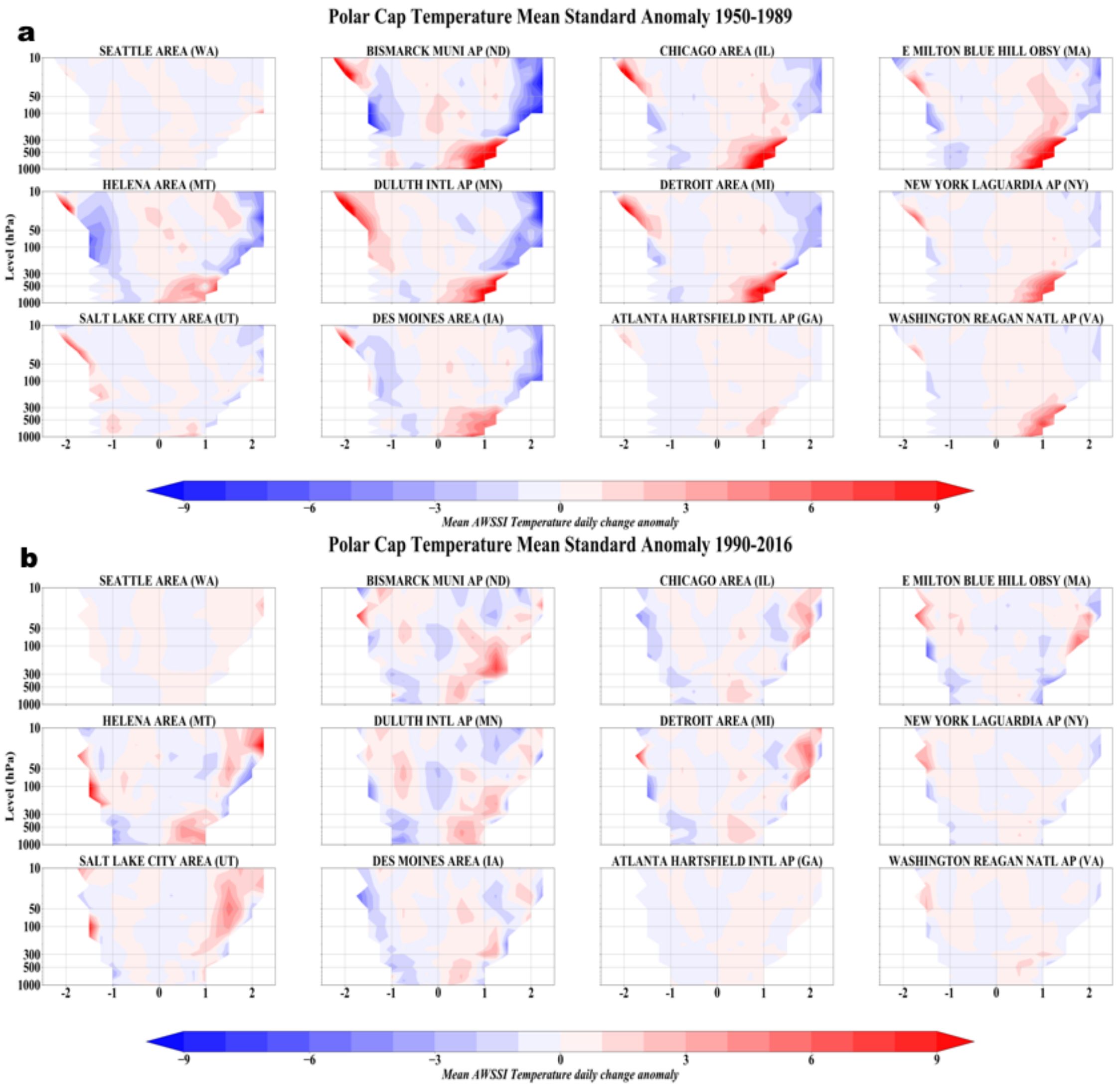

Supplementary Figure 3. Correlation between Arctic temperature anomalies (PCT) and severe winter weather (AWSSI) before and during period of AA. Same as Supplementary Figure 3 but for Arctic air temperatures **(PCT)**.

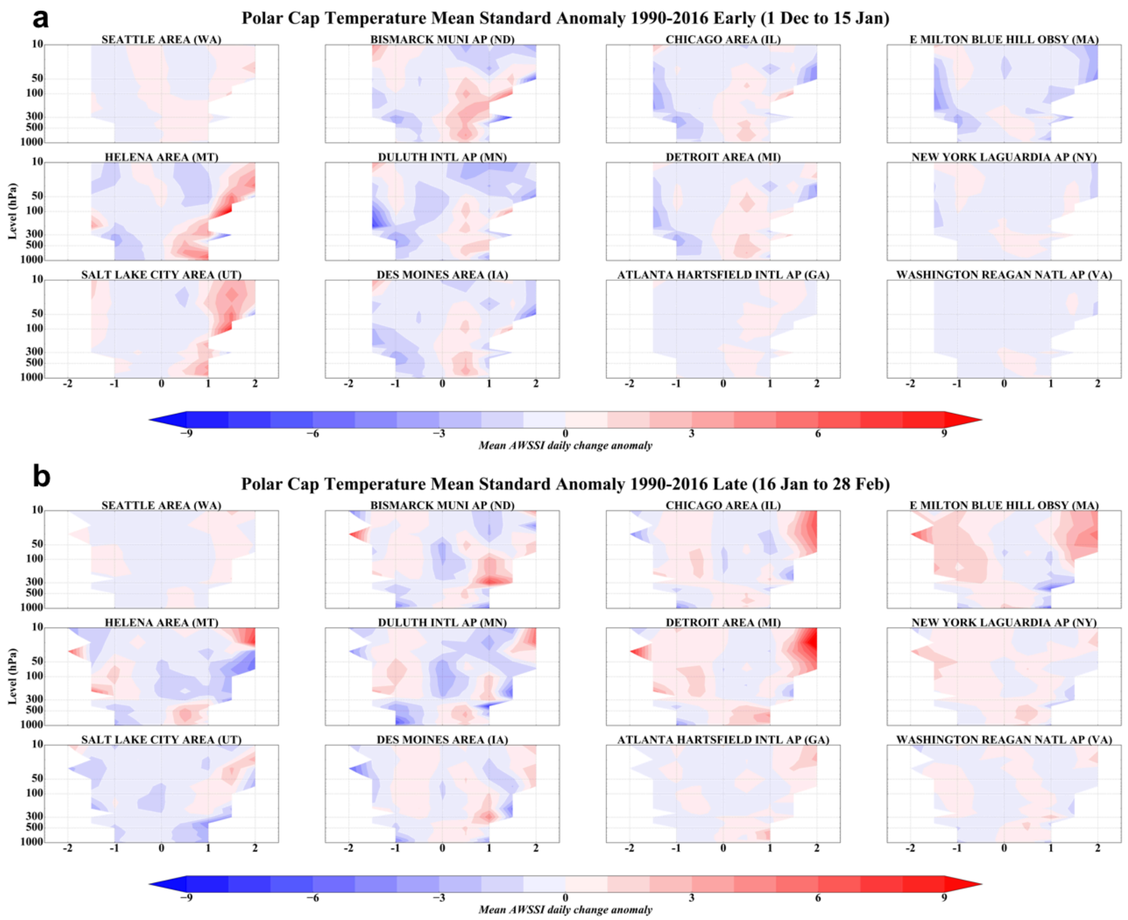

Supplementary Figure 4. Relationship between PCT and AWSSI is stronger in late compared to early winter in era of AA. Same as Supplementary Figure 3b but for **a** early winter (December -January 15) and **b** late winter (January 16 – February 28) in the era of Arctic amplification, especially in the stratosphere.

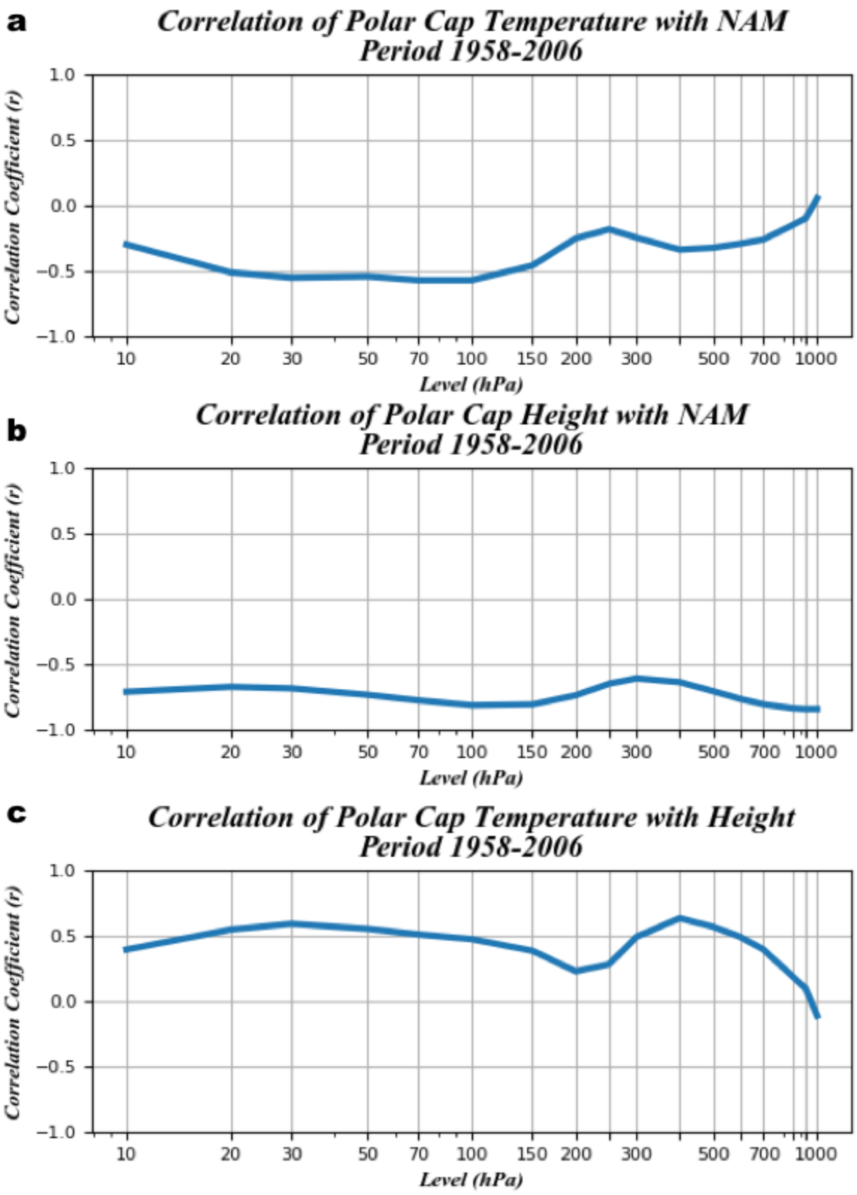

Supplementary Figure 5. Correlations between Arctic temperature, geopotential height surfaces, and the annular mode. The correlation between the PCH and the NAM from the surface to the mid-stratosphere (**a**), correlation between the PCT and NAM (**b**), and correlation between the PCT and PCH (**c**). Though the NAM and PCH are strongly correlated in the troposphere, the NAM and PCT are only weakly related throughout the troposphere.

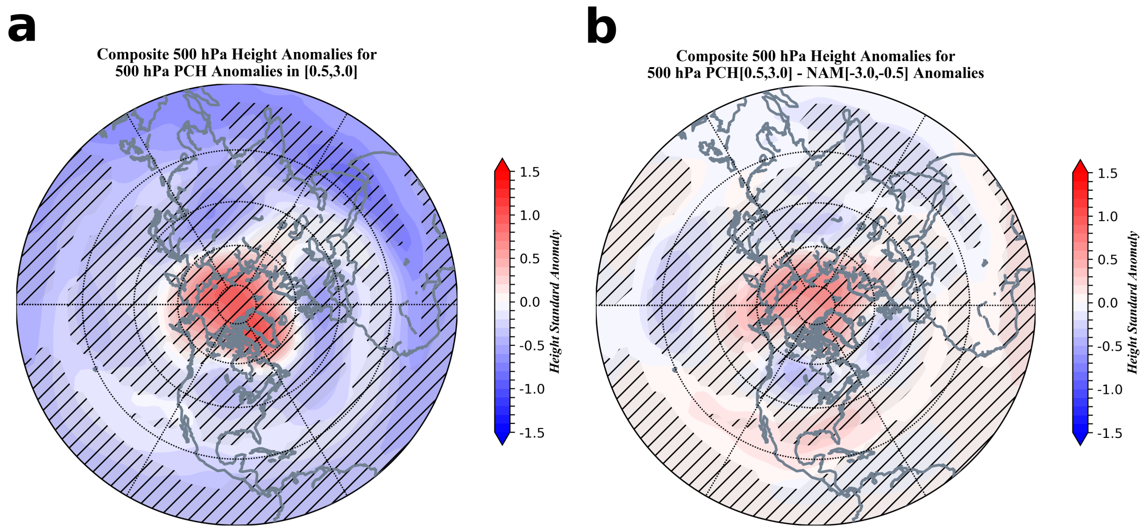

Supplementary Figure 6. Differences mid-troposphere geopotential heights between PCH and the NAM. **a** Northern Hemisphere 500 hPa geopotential height anomalies plotted for 500 hPa PCH binned on the intervals [0.5, 3.0] and **b** difference between 500 hPa geopotential height anomalies plotted for positive PCH and a negative NAM for all winters 1950-2016. Where difference was found to be statistically significant above 95% is hatched in light gray (e.g. [-0.5, -3.0] to [0.5, 3.0]).

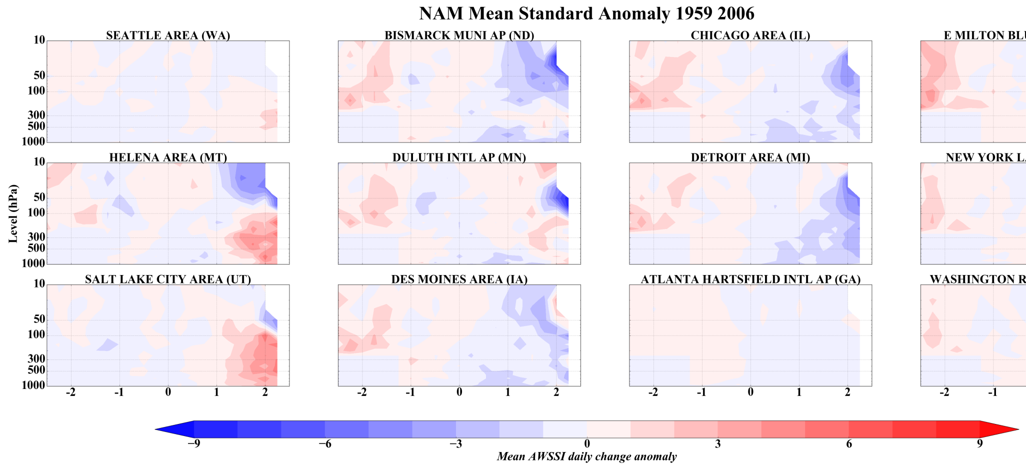

Supplementary Figure 7. Annular mode signal stronger in stratosphere while polar cap indices stronger in troposphere. Same as Figure 1 but using the NAM index instead of PCH/PCT.

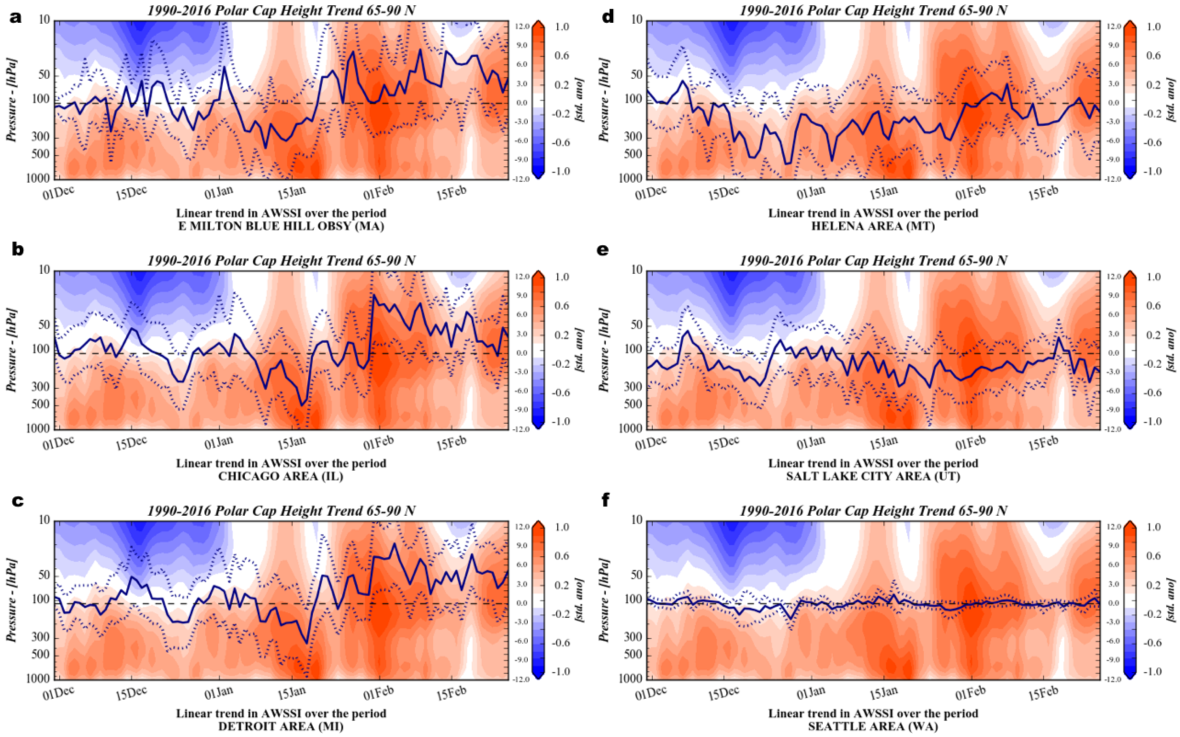

Supplementary Figure 8. Warming trend Arctic coincides with increased severe winter weather. The annual daily trend in PCH (shading) from the surface to mid-stratosphere (10 hPa) and the annual trend in daily change in AWSSI for three eastern US cities **a** Milton (near Boston), **b** Chicago, **c** Detroit and three western US cities **d** Helena, **e** Salt Lake City and **f** Seattle for the winters 1990/91-2015/16 multiplied by the total number of winters. Also included is the variability in the AWSSSI (±1 standard deviation) with the daily trend.
